# Health Literacy, Knowledge, and Risk Factors for Fatty Liver Disease among Asian American and Pacific Islanders and Latinos in Los Angeles

**DOI:** 10.31557/APJCP.2021.22.6.1737

**Published:** 2021-06

**Authors:** Minh P Nguyen, Aryana T Amoon, Lucia Lee, Vincent Chiang, Kourtney Nham, Aleck Q. Sun, Matthew Ji, Phillip Sundin, Roshan Bastani, Yvonne N Flores

**Affiliations:** 1 *University of California, Los Angeles (UCLA), Los Angeles, CA*, *United States. *; 2 *UCLA Center for Cancer Prevention and Control Research, Fielding School of Public Health and Jonsson Comprehensive Cancer Center, Los Angeles, CA, United States. *; 3 *Department of Biostatistics, UCLA Fielding School of Public Health, Los Angeles, CA, United States. *; 4 *UCLA Department of Health Policy and Management and UCLA Kaiser Permanente Center for Health Equity, Fielding School of Public Health, Los Angeles, CA, United States. *; 5 *Unidad de Investigación Epidemiológica y en Servicios de Salud, Morelos, Instituto Mexicano del Seguro Social, Cuernavaca, Morelos, CP México, United States. *

**Keywords:** Fatty liver disease, health literacy- knowledge, risk factors, Asian American and Pacific Islanders, Latinos

## Abstract

**Background:**

Fatty liver disease (FLD) is associated with increased risk for hepatocellular carcinoma (HCC) and is associated with rising rates of diabetes and obesity. The prevalence of FLD is rising among Asian American and Pacific Islanders (AAPIs) and Latinos. This study examined health literacy, knowledge, and risk factors for FLD among AAPIs and Latinos in Los Angeles.

**Methods:**

Data from in-person interviews and clinical measures (body mass index (BMI), body fat percentage, and blood pressure) were obtained from adults aged 18-82 years at four health fairs from November 2018 to March 2019. Interviews assessed knowledge about FLD, access to health resources, and satisfaction with current physician. Correct responses to knowledge questions were summed to generate a FLD knowledge score. Linear regression models were used to examine the association between knowledge score and age, sex, and race/ethnicity.

**Results:**

A total of 102 subjects were AAPI and 33 were Latino. Over 65% of participants had heard of FLD but demonstrated limited knowledge about FLD. Only 24% of subjects reported receiving FLD resources in their preferred language. Most subjects failed to identify several risk factors and key symptoms of FLD. Mean knowledge score for subjects who had heard of FLD was 7.58 (95% CI 7.15-8.01) out of a possible 16 points, and for those who had not who had not heard of FLD it was 5.71 (5.00-6.42) (p<0.0001).

**Conclusions:**

A lack of culturally competent resources and effective communication strategies between physicians and patients regarding FLD contributes to a lower awareness about the increased risk of FLD among AAPIs and Latinos. Future studies should investigate optimal methods to educate these communities about FLD and its associations with HCC.

## Introduction

Health literacy is defined as “the degree to which individuals have the capacity to obtain, process, and understand basic health information and services needed to make appropriate health decisions” (Ratzan et al., 2000). Low health literacy is associated with poorer access to care and health outcomes (Berkman et al., 2011). Access to health resources is also dependent on education level, quality of patient-physician interactions, and language discrepancy of health resources (Becerra et al., 2015, 2017). Individuals with low English proficiency are less likely to obtain screening for diseases such as cancer, which increases their risk of poor outcomes (Sentell and Braun, 2012; Genoff et al., 2016). Compared to Latinos in the United States (U.S.) who are English proficient, Latinos with poor English proficiency have an almost 3-fold higher risk of low health literacy (Sentell and Braun, 2012). 

Recent studies indicate that Asian Americans and Pacific Islanders (AAPIs) also exhibit low levels of health literacy compared to non-Hispanic Whites (Whites) (Browning et al., 2004). Within the AAPI community, health literacy is associated with sex, socioeconomic status, English language proficiency, and country of birth (U.S. vs other) (Sentell and Braun, 2012; Becerra et al., 2015). These findings support the need for more research on how health literacy issues are affecting the health and well-being of the Latino and AAPI communities, especially with regard to increasingly common conditions, such as fatty liver disease (FLD). 

FLD is a condition in which fat accumulates in the liver, beyond what is considered normal (>5%). The two primary types of FLD are non-alcoholic (NAFLD) and alcohol-related (AFLD). Left untreated, both can lead to complications of the liver and death due to cirrhosis or cancer (Chalasani et al., 2018). NAFLD is associated with obesity and type 2 diabetes (El-serag et al., 2004). Other established risk factors include dyslipidemia, hypertension, and metabolic syndrome (Farrell and Larter, 2006). Numerous studies have reported an association between FLD and an increased risk of hepatocellular carcinoma (HCC), strongly suggesting FLD as a risk factor for the disease along with diabetes and obesity (White et al., 2012). 

**Figure 1:**
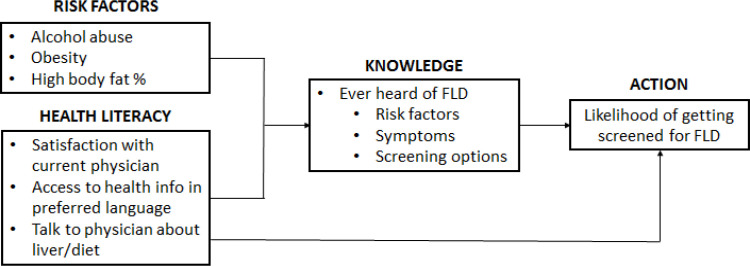
The Role of Risk Factors and Health Literacy of Fatty Liver Disease on Likelihood of Screening

AFLD affects an estimated 4.3% of the U.S. population (Wong et al., 2019), but alcoholic liver diseases account for 48% of deaths due to cirrhosis, the 12th leading cause of death (Yoon and Chen, 2016). The primary risk factor for AFLD is excessive alcohol consumption, which is linked to numerous chronic liver conditions (Hashimoto et al., 2004). Blood tests targeting liver-damage specific biomarkers such as alanine aminotransferase (ALT), as well as non-invasive tools such as ultrasound and elastography, are examples of tests that can be performed to screen for suspected FLD, although biopsy is the gold standard for diagnosis (Rinella, 2015) . Figure 1 describes the relationship between specific risk factors, health literacy, knowledge of FLD and the likelihood of getting screened for this disease.

NAFLD is the leading cause of chronic liver disease and the fastest growing cause of cirrhosis in the U.S. (Kanwal et al., 2018). The prevalence of non-alcoholic steatohepatitis (NASH), a more severe form of NAFLD, is more common among Latinos (45%), than in Whites (32%) or Blacks (20%) (Rich et al., 2018). Studies in Asia have revealed a significant rise in FLD prevalence, which has been associated with an increasingly urban and sedentary lifestyle (Fan et al., 2007). Social changes due to industrialization or immigration to post-industrialized nations have led to greater inactivity and a lower quality diet. AAPIs also have a higher prevalence of insulin resistance, metabolic syndrome, and dyslipidemia (Palaniappan et al., 2011). Additionally, while AAPIs generally have a lower Body Mass Index (BMI) and reduced obesity rates, they are still at risk of developing FLD due to a higher body fat percentages (Wong and Ahmed, 2014).

The aim of this pilot study was to assess health literacy, knowledge, and risk factors related to FLD, among a sample of AAPI and Latinos who attended health fairs in Los Angeles. We conducted in-person interviews and collected biometric data obtained as part of a community-based, observational study, to determine if participants with a lower knowledge of FLD risk factors are at higher risk of developing this disease. 

## Materials and Methods

*Study Population*

AAPI and Latino adults between the age of 18 and 82 years were invited to complete a brief survey while attending health fairs in Los Angeles County from November 2018 to March 2019. A convenience sampling approach was used to inform 498 individuals about the study and of these, 373 agreed to participate. A total of 135 participants completed both the in-person survey and biometric screening. Prior to data collection, informed consent was obtained from participants. Potential subjects were provided with detailed information about the purpose of the study, procedures, potential risks and benefits, confidentiality, and the voluntary nature of their participation. 

*Data Collection Activities*

The study survey was designed to determine knowledge of FLD risk factors, and potential areas for health literacy improvement, including access to health resources in their language of choice, and quality of patient-physician interactions. Age, race/ethnicity, primary language, sex, marital status, educational level, and employment status were also obtained. Interviews were conducted in a private space by student researchers in English, Mandarin, or Spanish based on language preference, and took approximately 15 minutes to complete. All student researchers attended a training led by study coordinators on questionnaire administration. Students proficient in Spanish or Mandarin were trained in the translation of the questions into either language, and all researchers were informed about the importance of patient confidentiality in compliance with UCLA IRB standards (UCLA IRB# 19-000326). Responses to questionnaires administered at the November 2018 health fairs were recorded on paper, while questionnaires completed in February and March 2019 were entered into a password-protected electronic format. No personal identifying information was collected. Study subjects did not receive any financial compensation for their participation, but were offered the option to have their BMI, body fat percentage, and blood pressure measured. The health screenings activities were conducted in English by default, but translation services were provided if requested. The health screenings were completely voluntary, and not all subjects received them. Health screening data were linked to survey responses via numerical codes. 

*Study Variables*

Socio-demographic Information. Age, sex, education status, and annual income.

FLD Knowledge. Participants were asked to agree or disagree with 16 statements in three domains: (1) Risk Factor Knowledge, (2) FLD Symptom Knowledge, and (3) Screening Knowledge, based on previously published research (Expert Panel on the Identification, Evaluation, and Treatment of Overweight in Adults, 1998; DeWalt et al., 2004; El-serag et al., 2004; Farrell and Larter, 2006; Fan and Farrell, 2009). 

Healthcare Services. Self-reported information was also collected about health insurance, access to and use of healthcare services, satisfaction with current physician, participant’s ability to obtain health information in their preferred language, receipt of informational resources for FLD in their preferred language, communicating with a physician about their liver or their diet, likelihood of receiving FLD screening in the future, and how participants would prefer to receive information and resources regarding liver health. 

Alcohol Consumption. Participants self-reported as Non-Drinker or Current Drinker, and the latter were asked to indicate how frequently they consume 5 or more drinks in one day.

Blood Pressure. Blood pressure was measured using electronic blood pressure cuffs. Normal blood pressure is defined by the American Heart Association as <120 mmHg systolic blood pressure (SBP) over <80 mmHg diastolic blood pressure (DBP); prehypertensive as 121-<130/<80 SBP/DBP, and hypertensive as ≥130/≥80 SBP/DBP (Understanding Blood Pressure Readings).

Body Mass Index (BMI). Height and weight were measured on-site. The following ranges were used for BMI: Normal (Asian: <23, Latino: <25), Overweight (Asian 23-27.4, Latino: 25-29.9), Obese (Asian: ≥27.5, Latino: ≥30) (Expert Panel on the Identification, Evaluation, and Treatment of Overweight in Adults, 1998).

Body Fat Percentage. Body fat percentage was estimated using handheld Omron HBF body fat monitors and participants were classified as Normal, Overweight, or Obese, by age and sex (Gallagher et al., 2000).

*Statistical analysis*

A descriptive analysis of various socio-demographic, clinical, and medical care characteristics was conducted by race/ethnicity and sex. Means and 95% confidence intervals were calculated for the following continuous variables, and percentages were determined for the categorical variables. Among those who had heard of FLD, we examined the proportion of respondents who correctly identified risk factors, symptoms, and screening options for FLD by race/ethnicity and sex. A knowledge score was calculated by summing the correct responses in the three knowledge domains, for a total of 16 points. Univariate and multivariable linear regression was performed with knowledge score as the outcome, and age, sex, and race/ethnicity as predictors. Analyses were performed using R version 3.6.1 and SAS version 9.3.

## Results

Table 1 presents the socio-demographic, clinical, and alcohol consumption information reported by the study participants, by race/ethnicity and sex. A total of 102 subjects were AAPI (34 males, 68 females) and 33 were Latino (14 males, 19 females). The mean age of the AAPI males was older than the Latino males (62.5 years vs. 48.6 years, respectively), but was similar for the AAPI and Latina females (57.3 years vs. 57.4, respectively). More than half of AAPI participants indicated an education level beyond high school. Between 35-42% of the AAPI respondents reported that their annual income was below $20,000 dollars. Over half of AAPI participants replied that they visited their doctor at least once a year, and over 70% stated that they had health insurance. Most Latino participants did not provide a response to these four items. A greater proportion of AAPI subjects (54%) had a normal BMI than Latino subjects (15%). However, using body fat percentage instead of BMI greatly increased the proportion of obese AAPI males (from 8.8% to 50% obese). Latino males reported the highest proportion of current drinking (35.7%), and similar binge drinking rates were observed among AAPI and Latino males.

Approximately 65% of all participants (n=88) had heard of FLD, including 66 AAPIs (64.7%) and 22 Latinos (62.9%). Table 2 reports the knowledge responses among those who had heard of FLD. Most subjects (68-85%) in each category accurately identified fatty food consumption as a risk factor for FLD. However, many respondents failed to identify lack of exercise (11-54%), increasing age (17-37%), and alcohol consumption as possibly associated with FLD (30-53%). Regardless of race/ethnicity and sex, most participants failed to identify any symptoms of FLD, with the exception of Latina females, who correctly identified abdominal pain (54%), swelling (62%), jaundice (62%), and nausea (45%). Most subjects (68-78%) correctly identified blood tests as a valid screening method and urine tests as not valid (78-89%). However, less than half of the participants responded that ultrasounds can also be used to screen for FLD. 

 The mean knowledge score for the 88 subjects who reported that they had heard of FLD was 7.58 (95% CI 7.15-8.01), out of a possible 16 points. For participants who had not heard of FLD (n=41), the score was 5.71 (95% CI 5.00-6.42). The difference between the two scores was statistically significant (p<0.0001). The results of a simple linear regression model, with knowledge score as the outcome, and age, sex, and race/ethnicity included as covariates, indicated that subjects who were older, male, and AAPI have lower knowledge scores than their respective counterparts, but these findings were not statistically significant.

Table 3 presents the participants’ responses regarding their access to and use of healthcare services, as well as satisfaction and communication with their healthcare provider. Between 64 and 90% of subjects indicated that they are satisfied or very satisfied with their current physician, and 38-64% reported that their ability to access health information is “easy.” Less than one-fourth of the participants (<24%) reported having received resources for FLD in their preferred language. More Latinos indicated that they had talked to their physician or dietician about their diet than AAPIs. Both AAPIs and Latinos stated that simpler terms and multilingual resources would help improve their understanding of liver health. Finally, Latino males were the most likely to consider FLD screening in the future (93%), with AAPI males as the most unlikely to get screened (29%).

**Table 1:** Comparison of Socio-Demographic Characteristics and Fatty Liver Disease Risk Factors in AAPI and Latinos in Los Angeles (n=135)

	AAPI*		Latino*	
	Male (n=34)	Female (n=68)	Male (n=14)	Female (n=19)
Age (years)				
Mean (95% CI)	62.5 (58.6-66.5)	57.3 (53.7-60.8)	48.6 (39.9-57.2)	57.4 (51.7-63.2)
< 60	11 (32.4%)	29 (42.6%)	11 (78.6%)	10 (52.6%)
≥ 60	23 (67.6%)	35 (51.5%)	3 (21.4%)	9 (47.3%)
No response	0 (0%)	4 (5.9%)	0 (0%)	0 (0%)
Education Status				
≤ High School	13 (38.2%)	24 (35.3%)	2 (14.3%)	3 (15.8%)
> High School	19 (55.9%)	40 (58.8%)	2 (14.3%)	2 (10.5%)
No response	2 (5.9%)	4 (5.9%)	10 (71.4%)	14 (73.7%)
Annual Income				
< $20,000	12 (35.3%)	29 (42.6%)	1 (7.1%)	2 (10.5%)
≥ $20,000	11 (32.4%)	13 (19.1%)	2 (14.3%)	1 (5.3%)
No response	11 (32.4%)	26 (38.2%)	11 (78.6%)	16 (84.2%)
Visit a Doctor Regularly (≥ Once per year)				
Yes	23 (67.6%)	39 (57.4%)	3 (21.4%)	4 (21.1%)
No	32 (32.4%)	29 (42.6%)	1 (7.1%)	1 (5.3%)
No response	0 (0%)	0 (0%)	10 (71.4%)	14 (73.7%)
Have Health Insurance				
Yes	27 (79.4%)	50 (73.5%)	3 (21.4%)	4 (21.1%)
No	6 (17.7%)	18 (26.5%)	1 (7.1%)	1 (5.3%)
No Response	1 (2.9%)	0 (0%)	10 (71.4%)	14 (73.7%)
BMI (kg/m^2^)				
Mean (95% CI)	25.7 (24.0-27.3)	23.6 (22.6-24.6)	27.5 (25.6-29.4)	28.2 (24.7-31.7)
Normal (Asian: <23, Latino: <25)	14 (41.2%)	42 (61.8%)	1 (7.1%)	4 (21.1%)
Overweight (Asian 23-27.4, Latino: 25-29.9)	16 (47.1%)	14 (20.6%)	9 (64.3%)	6 (31.6%)
Obese (Asian: ≥27.5, Latino: ≥30)	3 (8.8%)	3 (4.4%)	1 (7.1%)	4 (21.1%)
No response	1 (2.9%)	9 (13.2%)	3 (21.4%)	5 (26.3%)
Body Fat (%) ^†^				
Mean (95% CI)	28.0 (25.8-30.2)	33.3 (31.3-35.2)	27.3 (24.2-30.5)	38.7 (34.7-42.7)
Normal	5 (14.7%)	29 (42.6%)	0 (0.0%)	3 (15.8%)
Overweight	8 (23.5%)	17 (25.0%)	5 (35.7%)	3 (15.8%)
Obese	17 (50.0%)	12 (17.6%)	4 (28.6%)	6 (31.6%)
No response	4 (11.8%)	10 (29.4%)	5 (35.7%)	7 (36.8%)
Blood Pressure (SBP/DBP mmHg)				
Normal (<120/<80)	5 (14.7%)	27 (39.7%)	2 (14.3%)	4 (21.1%)
Prehypertensive (121-130/<80)	5 (14.7%)	4 (5.9%)	4 (28.6%)	3 (15.8%)
Hypertensive (≥130/≥80)	18 (52.9%)	18 (26.5%)	5 (35.7%)	9 (47.4%)
No response	6 (17.6%)	19 (27.9%)	3 (21.4%)	3 (15.8%)
Alcohol Consumption				
Non-Drinker	27 (79.4%)	48 (70.6%)	8 (57.1%)	15 (78.9%)
Current Drinker	5 (14.7%)	11 (16.2)%)	5 (35.7%)	4 (21.1%)
No response	2 (5.8%)	9 (13.2%)	1 (7.1%)	0 (0.0%)
Binge Drinker (≥5 drinks/day)¥				
≤ Once a month	3 (75.0%)	7 (63.6%)	4 (80.0%)	4 (100.0%)
≥ Once a week	1 (20.0%)	4 (36.4)	1 (20.0%)	0 (0.0%)
No response	1 (20.0%)	0 (0.0%)	0 (0.0%)	0 (0.0%)

AAPI, Asian American and Pacific Islander; CI, confidence interval; BMI, body mass index; SBP, systolic blood pressure; DBP, diastolic blood pressure; *Values reported as n (%) unless otherwise stated. Percents may not add to 100% in cases where subjects declined to respond. †Body fat percentages: Asian females 20-39 years, Normal (25.9-32.5%), Overweight (32.6-38.2%), Obese (≥38.3%); 40-59 years, Normal (26.5-33.0%), Overweight (33.1-38.8%), Obese (≥38.9%); 60-79 years, Normal (27.0-33.6%), Overweight (33.7-39.4%), Obese (≥39.5%). Asian males 20-39 years, Normal (13.8-20.4%), Overweight (20.5-26.1%), Obese (≥26.2%); 40-59 years, Normal (14.4-21.0%), Overweight (21.1-26.7%), Obese (≥26.8%); 60-79 years, Normal (14.9-21.5%), Overweight (21.6-27.3%), Obese (≥27.4%). Latina females 20-39 years, Normal (21.0-32.9%), Overweight (33.0-38.9%), Obese (≥39.0%); 40-59 years, Normal (23.0-33.9%), Overweight (34.0-39.9%), Obese (≥40.0%); 60-79 years, Normal (24.0-35.9%), Overweight (36.0-41.9%), Obese (≥42.0%). Latino males 20-39 years, Normal (8.0-19.9%), Overweight (20.0-24.9%), Obese (≥25.0%); 40-59 years, Normal (11.0-21.9%), Overweight (22.0-27.9%), Obese (≥28.0%); 60-79 years, Normal (13.0-24.9%), Overweight (25.0-29.9%), Obese (≥30.0%). ^¥^Proportions of Binge Drinkers were for those who reported that they were a Current Drinker in the Alcohol Consumption section

**Table 2:** Proportion of Correct Responses to Questions about Fatty Liver Disease Risk Factors, Symptoms, and Screening by Race/Ethnicity and Sex in People who have Previously Heard of FLD (n= 88)

Knowledge Item	AAPI (%)		Latino (%)	
	Males (n=19)	Females (n=47)	Males (n=9)	Females (n=13)
Risk Factor Knowledge				
Item 1. Fatty foods increase risk of FLD (agree)	68	74	78	85
Item 2. Lack of exercise increases risk of FLD (agree)	42	51	11	54
Item 3. Lack of rest increases risk of FLD (disagree)	63	83	89	69
Item 4. Religion/fate increases risk of FLD (disagree)	95	74	100	85
Item 5. Age increases risk of FLD (agree)	37	17	22	23
Item 6. Negative attitude increases risk of FLD (disagree)	74	72	100	69
Item 7. Alcohol increases risk of FLD (agree)	53	30	44	38
Symptom Knowledge				
Item 8. Stomach pain is a symptom of FLD (agree)	32	28	33	54
Item 9. Swelling is a symptom of FLD (agree)	16	21	22	62
Item 10. Coughing is a symptom of FLD (disagree)	95	98	100	92
Item 11. Jaundice is a symptom of FLD (agree)	16	17	22	62
Item 12. Nausea is a symptom of FLD (agree)	21	17	22	45
Item 13. Fatigue is a symptom of FLD (agree)	26	49	33	31
Screening Knowledge				
Item 14. Ultrasounds can be used to screen for FLD (agree)	37	43	0	46
Item 15. Blood tests can be used to screen for FLD (agree)	74	68	78	77
Item 16. Urine tests can be used to screen for FLD (disagree)	89	89	78	85
Mean knowledge score (95% CI)	7.42 (6.40-8.44)	7.34 (6.77-7.92)	7.33 (5.90-8.77)	8.85 (7.59-10.10)

FLD, fatty liver disease; AAPI, Asian American and Pacific Islander; CI, confidence interval; Proportions are out of people who answered the question; Knowledge score was calculated out of a total of 16 points.

**Table 3:** Proportion of Responses to Questions about Medical Care and Liver Ddisease Resources by Race/Ethnicity and Sex

	AAPI (%)		Latinos (%)	
	Male (n=34)	Female (n=68)	Male (n=14)	Female (n=19)
Satisfaction with Current Physician				
Very Satisfactory	24	21	21	11
Satisfactory	53	60	43	79
Non-Satisfactory	6	3	14	5
No response	17	15	21	5
Ability to Access Health Information				
Easy	50	38	64	53
Neutral	29	43	21	37
Difficult	9	13	14	5
No response	12	4	0	5
Received Resources for FLD in Preferred Language				
Yes	24	19	21	21
No/Unsure	62	62	71	68
No response	15	19	7	11
Talked to Physician about Liver				
Yes	27	21	64	16
No	59	63	21	84
No response	15	16	14	0
Talked to Physician/Dietician about Diet				
Yes	35	26	43	47
No/Unsure	65	74	57	53
No response	0	0	0	0
What would improve your understanding of Liver Health*				
Multilingual Resources	38	28	21	37
Simpler Terms	21	34	64	79
More Pictures	9	19	43	47
Shorter Content	6	4	43	32
None of the above	9	15	0	0
Likelihood of FLD screening				
Likely	41	15	93	47
Neutral	21	38	7	16
Unlikely	29	13	0	16
No response	9	34	0	21

AAPI, Asian American and Pacific Islander; FLD, fatty liver disease; Percentages may not add up to 100% due to rounding and in cases where subjects declined to respond; *Participants could select any number of the possible choices

## Discussion

This study compared the risk factors, health literacy, and knowledge regarding FLD, an important risk factor for HCC, among a sample of AAPIs and Latinos in Los Angeles County. BMI and body fat percentage measurements indicate that Latinos have greater rates of overweight or obesity in general. Combined with the higher self-reported rates of alcohol consumption we observed among Latinos, our findings suggest that Latinos have greater increased risk for FLD than the AAPI participants. Although there were fewer Latino respondents than AAPI in our study, these results support those reported by our research team and other investigators, who found higher rates of obesity and alcohol consumption among U.S. Latinos (Flores et al., 2012, 2018). Studies conducted in China indicate that the prevalence of FLD, particularly obesity-correlated NAFLD, which affects approximately 19.6% of the historically lean mainland population, has doubled in the last decade (Fan and Farrell, 2009). Elevated rates of overweight and obesity were observed in both race/ethnicity groups, with a particularly high proportion of obesity among Asian males (50%), as measured by body fat percentage. 

Although two-thirds of the participants reported that they had heard of FLD, their responses to specific questions did not indicate substantial knowledge regarding the disease. Identification of risk factors was inconsistent between race/ethnicity and sex groups. Most subjects correctly identified fatty food consumption, but missed lack of exercise, which also affects likelihood of obesity, the major risk factor for NAFLD. The low rates of correctly identifying alcohol consumption as a risk factor suggest a lack of understanding of AFLD. Knowledge of symptoms was poor across the groups with Latinas as the most likely to correctly identify abdominal pain, swelling, jaundice, and nausea as FLD symptoms. Blood tests were the most commonly recognized method of screening for FLD, but ultrasounds were missed by more than half of subjects. Knowledge results were consistent with a New York study, where only 20% of respondents had ever heard of cirrhosis (Ghevariya et al., 2014) and a Hong Kong study, where less than 50% of respondents who had heard of NAFLD knew of its clinical manifestations (Leung et al., 2009). 

Linear regressions confirmed these results. From a total of 16 points, the average score ranged from 7.3 among Latino males to 8.9 among Latino females, with an average of 7.35 points among male and female AAPIs. Participants who were simply guessing scored an average of 5.71 points. Overall, males had lower knowledge scores than females and older age was associated with lower knowledge scores, but these results were not statistically significant, possibly due to small sample sizes. Health literacy is generally lower among older adults (Kutner et al., 2006), which may explain why the AAPI subjects had a lower knowledge of the risk factors, symptoms and screening tests for FLD, as over half of them were at least 60 years old. Overall, our results suggest a general lack of FLD knowledge regardless of race/ethnicity, age, or sex, with many subjects demonstrating both increased risk and a lack of preparedness to recognize and prevent the disease. These findings emphasize the need for increased communication between healthcare providers and their patients regarding FLD, and for more educational resources to improve patients’ awareness of the lifestyle changes required to mitigate risk. 

Latinos males were more likely to respond that they had talked to their physicians about their liver health than others, which may be due to higher reported rates of drinking (36%). However, all groups indicated low rates of speaking with their physicians regarding their diet, and few had discussed their liver health with their physicians (16-27%). Combined with high rates of overweight/obese BMI and body fat percentage, as well as an inadequate knowledge regarding FLD, lack of communication with healthcare providers points to a grossly inadequate response to help address the high risk of FLD in these communities. Lapses in effective communication, as well as lack of knowledge of the disease, may lead in delays in or negative perceptions of screening and treatment (Burnham et al., 2014).

The primary limitation of this pilot study is the relatively small sample size, particularly for Latinos, and the low response rate among some of the Latino participants. A previous study found a low knowledge of risk factors for liver disease among Latinos living in Los Angeles County (Flores et al., 2012). Our study expanded upon those results by including the less studied population of AAPI living in Los Angeles and found a similar lack of knowledge. Furthermore, despite the overall low sample size, this study yielded a high response rate to most questions. Since many participants indicated that English was not their preferred language, our study participants were likely representative of the AAPI and Latino populations in Los Angeles who have limited English proficiency. Another limitation was the lack of questions to assess participant acculturation. Defined as the process of adopting the beliefs and behaviors of another cultural group, acculturation has proven to be a useful variable when studied in relation to healthcare access and outcomes, particularly among Latinos living in the United States (Lara et al., 2004). Additionally, interviews are inherently prone to several types of response bias, including question order bias, where the order of the questions affects how people respond. Responses to other questions, such as the alcohol consumption question, could have been influenced by social desirability bias, where respondents answer in a way that makes them appear most favorable. Simple non-responses can also affect results if responses are not missing completely at random. 

Future studies should include a larger sample of both AAPI and Latino participants that will allow for more robust comparisons. This is particularly important due to the lack of research regarding the knowledge and risk of FLD among AAPI groups, which is not well documented or understood. Further examination in the form of newly devised questions is also required to investigate the effect of acculturation on FLD awareness, prevalence, and morbidity in these communities. A previous study reported an association of Non-Alcoholic Fatty Liver Disease risk factors with acculturation, although they saw no association with actual risk of the disease (Balakrishnan et al., 2017). The relationship between acculturation and risk for liver disease among AAPI is less well understood, although surprisingly it has been positively associated with alcohol use (a risk factor for FLD), further necessitating both inclusion of acculturation as a variable and more study of AAPI populations (Lui and Zamboanga, 2018). 

This is the first study to assess health literacy and knowledge of FLD risk factors in both AAPI and Latino communities in Los Angeles County. The results of our study indicate that health literacy, particularly regarding FLD, is low in these populations which tend to have higher rates of the disease. Our findings support those of other studies, which report that certain ethnic subgroups, such as Latino males in particular, could be at greater risk of FLD, and consequently for HCC (Flores et al., 2012, 2018). Increased education may serve as a preventative measure. Simpler terms and translation of educational materials into preferred languages were self-reported as potentially helpful methods to increase awareness and knowledge about the risk factors, symptoms, and screening options for FLD. Healthcare providers should take more active roles in discussing the importance of diet, exercise and liver health with their AAPI and Latino patients who may be at risk for FLD. 

## Author Contribution Statement

All authors contributed to the study conception and design. Data collection was performed by Minh P. Nguyen, Lucia Lee, Vincent Chiang, Kourtney Nham, Aleck Qihao Sun, and Matthew Ji. Data analysis and interpretation were performed by Phillip Sundin, Yvonne N. Flores, and Minh P. Nguyen. Drafting and revision of the manuscript was performed by Minh P. Nguyen, Aryanna T. Amoon, and Yvonne N. Flores. All authors read and approved the final manuscript.
